# Supplementary material for: Structural basis of ion uptake in copper-transporting P1B-type ATPases
Source: Nat Commun. 2022 Aug 31;13:5121. doi: 10.1038/s41467-022-32751-w (PMC9433437; doi:10.1038/s41467-022-32751-w)

SUPPLEMENTARY INFORMATION

STRUCTURAL BASIS OF ION UPTAKE IN COPPER-TRANSPORTING P_{1B}-TYPE ATPASES

Nina Salustros[1], Christina Grønberg[1], Nisansala Abeyrathna[2], Pin Lyu[1,3], Fredrik Orädd[4], Kaituo Wang[1], Magnus Andersson[2], Gabriele Meloni[2] & Pontus Gourdon[1,5,†]

[1] Department of Biomedical Sciences, Copenhagen University, Maersk Tower 7-9, Nørre Allé 14, DK-2200 Copenhagen, Denmark

[2] Department of Chemistry and Biochemistry, The University of Texas at Dallas, 800 W Campbell Rd., Richardson, TX 75080, USA

[3] Department of Biology, University of Copenhagen, Universitetsparken 13, DK-2100 Copenhagen, Denmark

[4] Department of Chemistry, Umeå University, Linneaus Väg 10, SE-901 87 Umeå, Sweden

[5] Department of Experimental Medical Science, Lund University, Sölvegatan 19, SE-221 84 Lund, Sweden

[†] Correspondence: pontus@sund.ku.dk (PG)

Supplementary Table 1: Data collection and refinement statistics. Statistics for the highest-resolution shell are shown in parentheses.

	Native $CuSO_4$	Cu Edge $CuSO_4$	Native apo
**PDB-ID:**	**7R0I**	**7R0H**	**7R0G**
**Wavelength**	1	1.37	1
**Resolution range**	45.65 - 2.705 (2.802 - 2.705)	44.96 - 3.31 (3.429 - 3.31)	46.13 - 4.011 (4.154 - 4.011)
**Space group**	F 2 2 2	F 2 2 2	C 1 2 1
**Unit cell**	129.526 150.098 218.906 90 90 90	130.122 151.115 219.211 90 90 90	128.194 226.056 111.106 90 134.062 90
**Total reflections**	195754 (14687)	617768 (57539)	153786 (15858)
**Unique reflections**	29155 (2798)	16266 (1599)	18264 (1863)
**Multiplicity**	6.7 (5.2)	38.0 (36.0)	8.4 (8.5)
**Completeness (%)**	99.63 (97.39)	99.63 (97.85)	93.95 (80.06)
**Mean I/sigma(I)**	19.66 (1.10)	21.49 (1.96)	6.88 (0.44)
**Wilson B-factor**	78.05	113.09	191.11
**R-merge**	0.0684 (1.283)	0.1612 (1.792)	0.2057 (3.855)
**R-meas**	0.07416 (1.423)	0.1634 (1.817)	0.219 (4.099)
**R-pim**	0.02837 (0.6053)	0.02641 (0.3003)	0.07477 (1.386)
**CC1/2**	1 (0.482)	1 (0.84)	0.996 (0.145)
**CC***	1 (0.806)	1 (0.956)	0.999 (0.503)
**Reflections used in refinement**	29141 (2794)	16255 (1596)	17902 (1530)
**Reflections used for R-free**	1999 (192)	1625 (159)	1788 (150)
**R-work**	0.2254 (0.4059)	0.2140 (0.3497)	0.2626 (0.4074)
**R-free**	0.2566 (0.4260)	0.2509 (0.3923)	0.3114 (0.4159)
**CC(work)**	0.934 (0.610)	0.905 (0.826)	0.927 (0.276)
**CC(free)**	0.944 (0.514)	0.846 (0.723)	0.774 (0.409)
**Number of non-hydrogen atoms**	4931	4928	9841
**macromolecules**	4929	4927	9841
**ligands**	2	1	0
**solvent**	0	0	0
**Protein residues**	654	654	1306
**RMS(bonds)**	0.011	0.011	0.005
**RMS(angles)**	1.27	1.40	1.06
**Ramachandran favored (%)**	93.87	92.79	94.32
**Ramachandran allowed (%)**	5.83	7.06	5.15
**Ramachandran outliers (%)**	0.31	0.15	0.54
**Rotamer outliers (%)**	2.88	0.00	0.10
**Clashscore**	8.12	10.31	17.35
**Average B-factor**	93.39	115.43	203.75
**macromolecules**	93.40	115.38	203.75
**ligands**	85.02	358.81	

Supplementary Fig. 1.

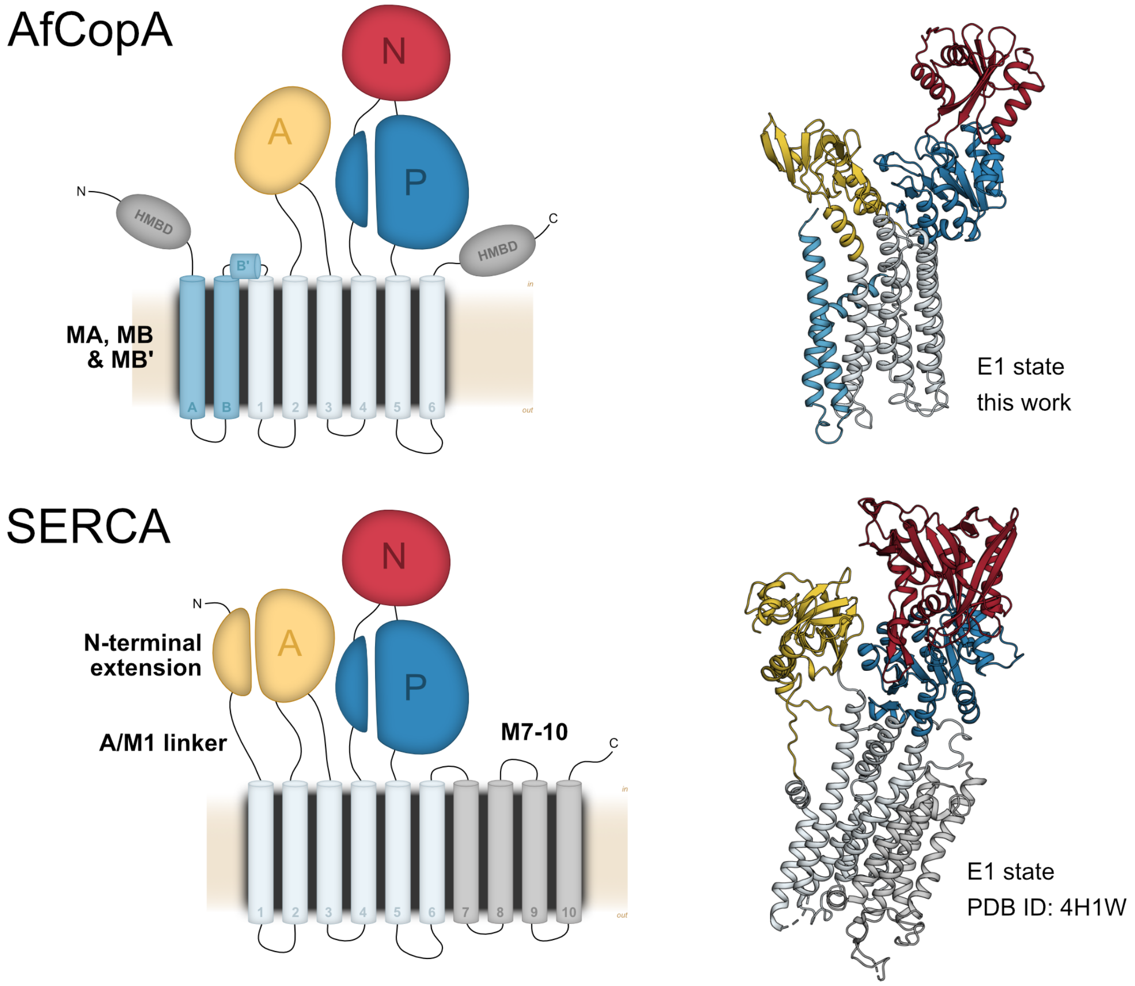

Supplementary Fig. 1. Topology of CopA compared to SERCA. The proteins are colored as in Fig. 1. The P_{1B-1}-ATPase AfCopA has a N- and C-terminal heavy metal-binding domain (HMBD), respectively, both of which were removed from the structurally determined construct (AfCopAΔNΔC). CopA harbors eight transmembrane helices MA, MB and M1-6. The P_{2A}-ATPase SERCA has ten transmembrane helices M1-10. MA and MB are absent in SERCA, but an N-terminal A-domain extension in present. The determined AfCopA structure and the corresponding E1 state of SERCA (PDB-ID 4H1W) are shown as cartoons.

Supplementary Fig. 2.

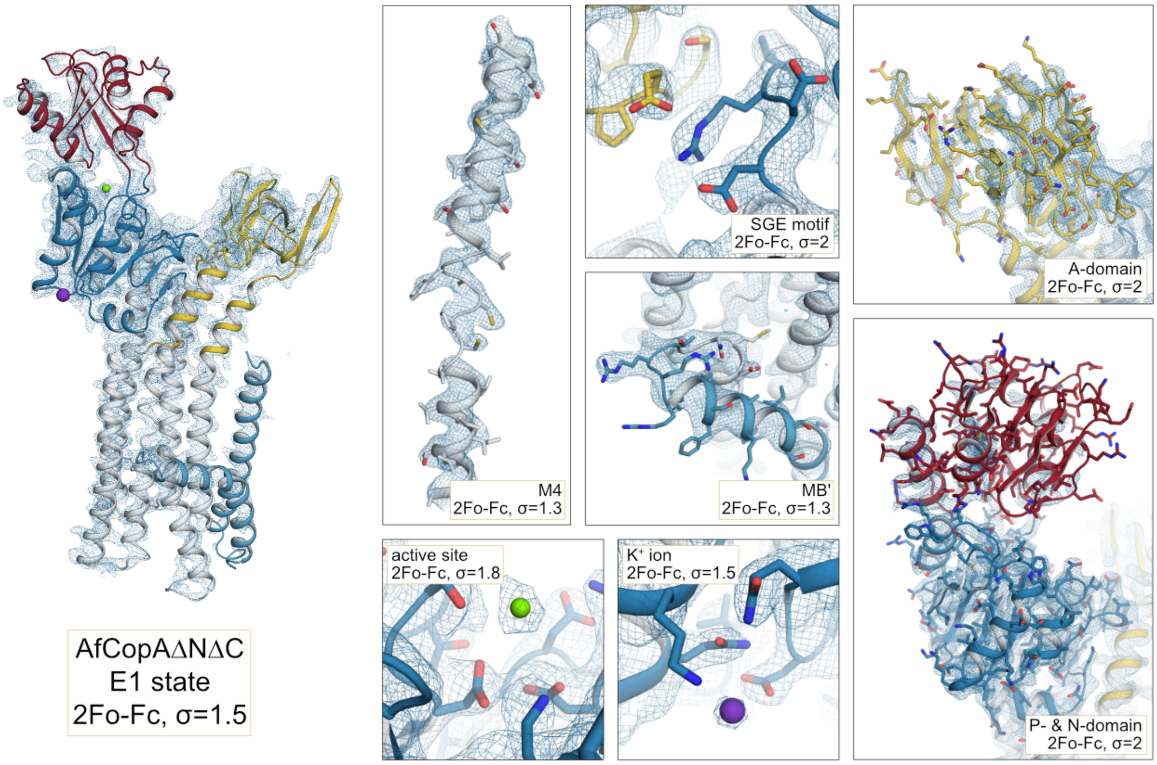

Supplementary Fig. 2. Map quality. The final 2Fo-Fc map derived from the 2.7 Å data set of the obtained AfCopA E1 state is shown in blue at the indicated contour levels.

Supplementary Fig. 3.

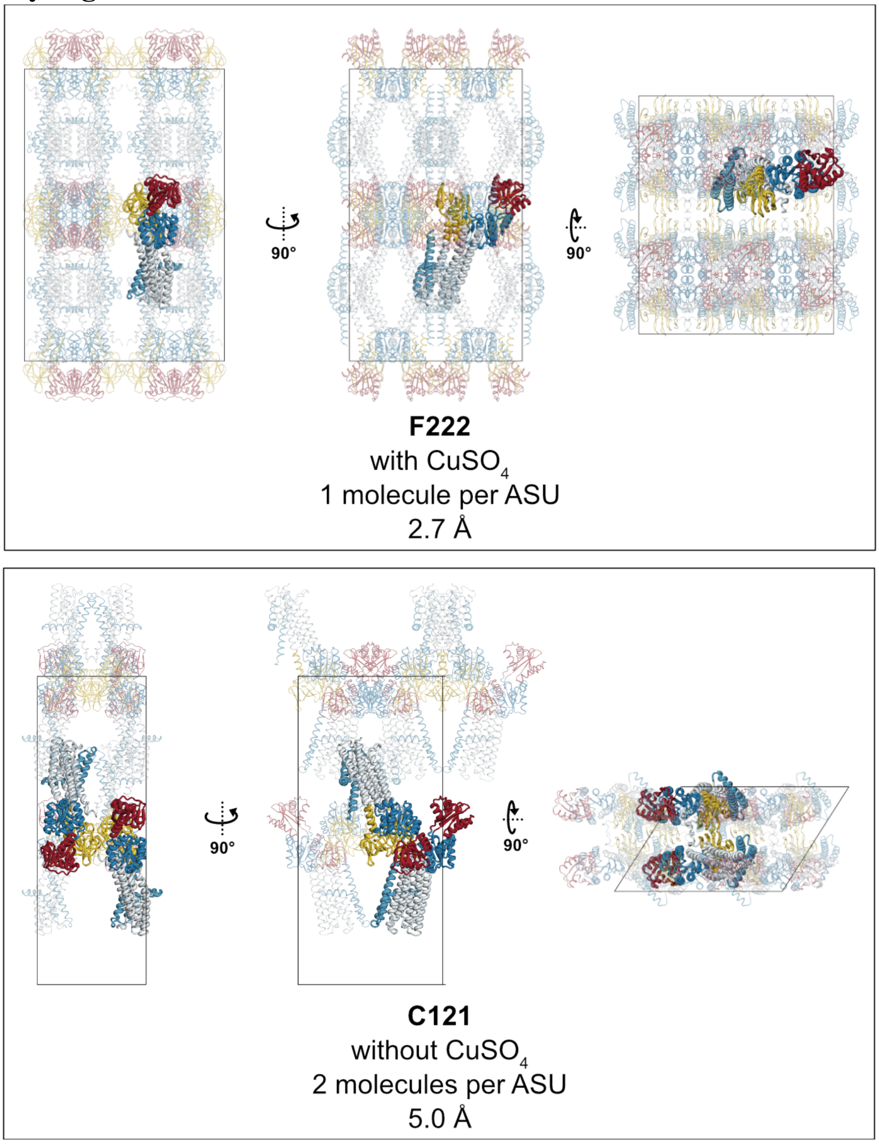

Supplementary Fig. 3. Crystal packing. Crystals grown in the presence and absence of $CuSO_4$ arranged in F222 and C121 space groups, respectively. The highest resolutions recovered for these crystal forms were 2.7 and 5.0 Å, respectively. The asymmetric unit (ASU) is highlighted, and symmetry-related molecules are shown in transparent.

Supplementary Fig. 4.

SERCA state	SERCA PDB-ID	AfCopA	LpCopA 4BBJ	LpCopA 3RFU
E1	4H1W	10.227	10.071	11.882
$[Ca]_2$ E1	2C9M	13.161	11.534	15.270
$[Ca]_2$ E1	1SU4	14.198	11.050	14.095
$[Ca]_2$ E1·ATP	3N8G	**9.177**	10.392	11.886
$[Ca]_2$ E1-ADP:AlF4	1T5T	9.189	10.492	11.971
$[Ca]_2$ E1P-ADP	1T5S	9.181	10.482	12.073
$[Ca]_2$ E1P:ADP	3BA6	9.301	10.426	11.838
E2P	3B9B	15.314	**2.192**	3.857
E2-P	3N5K	15.364	3.123	2.768
E2:Pi	3FGO	15.435	3.013	**2.679**
E2	3NAL	12.748	5.326	6.162

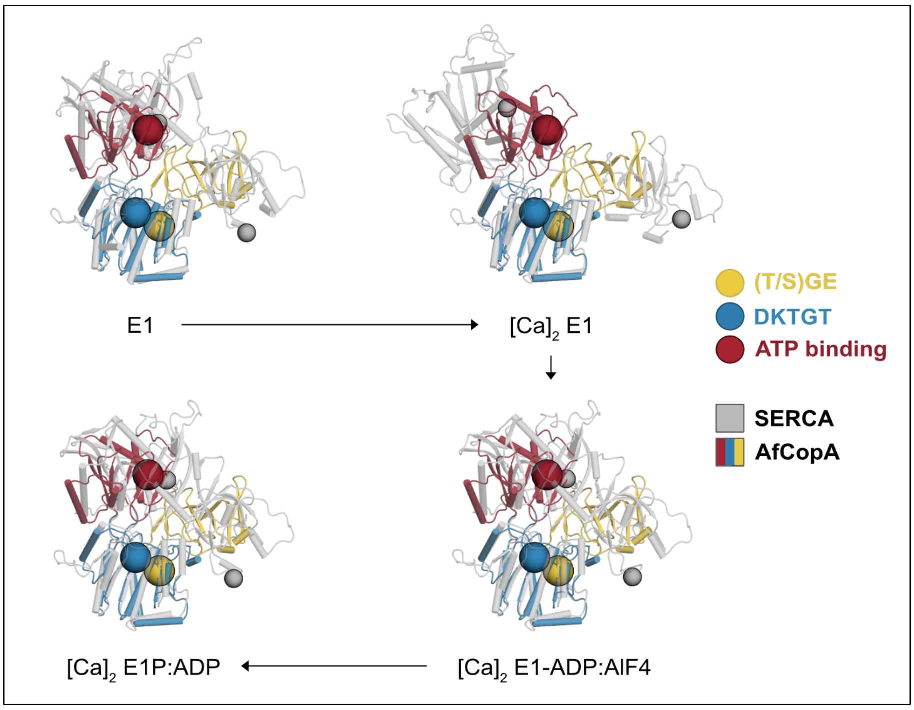

Supplementary Fig. 4. Structure-based alignments of the obtained AfCopA structure to available structures of SERCA. The table lists root-mean-square deviations (RMSD) based on alignments of the soluble A-, P- and N-domains. Surprisingly, the determined E1 conformation of AfCopA displays highest similarity to the $[Ca]_2$ E1-ADP:AlF state of SERCA. The figure illustrates alignments of different SERCA E1 conformations to the determined AfCopA structure based on the P-domain only. The soluble A-, P- and N-domains are displayed, and the catalytic aspartate, (T/S)GE motif of the A-domain (yellow) and ATP binding pocket in the N-domain (red) are shown as spheres. Notably, the arrangement of the soluble domains in the determined AfCopA structure does not match any of the available structures of SERCA.

Supplementary Fig. 5.

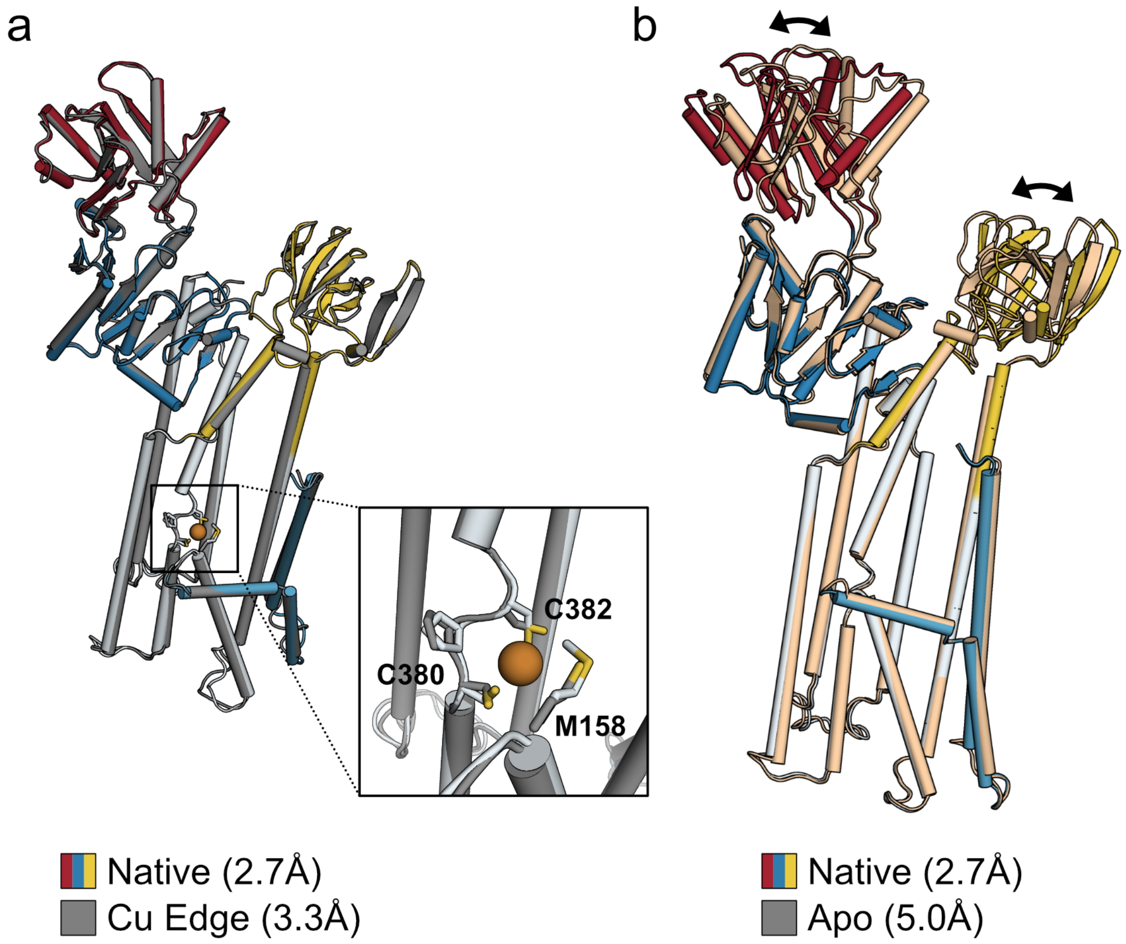

Supplementary Fig. 5. Alignment of the determined AfCopA structures. a High-resolution data set collected at 1 Å wavelength (Native, red/blue/yellow) aligned to the P-domain of the model derived from the data set collected at the Cu Edge (1.37 Å, dark grey). Inset shows residues around the detected copper binding site. **b** Comparison of high-resolution and the low-resolution structure crystallized in the absence of copper and nucleotides (apo, light grey).

Supplementary Fig. 6.

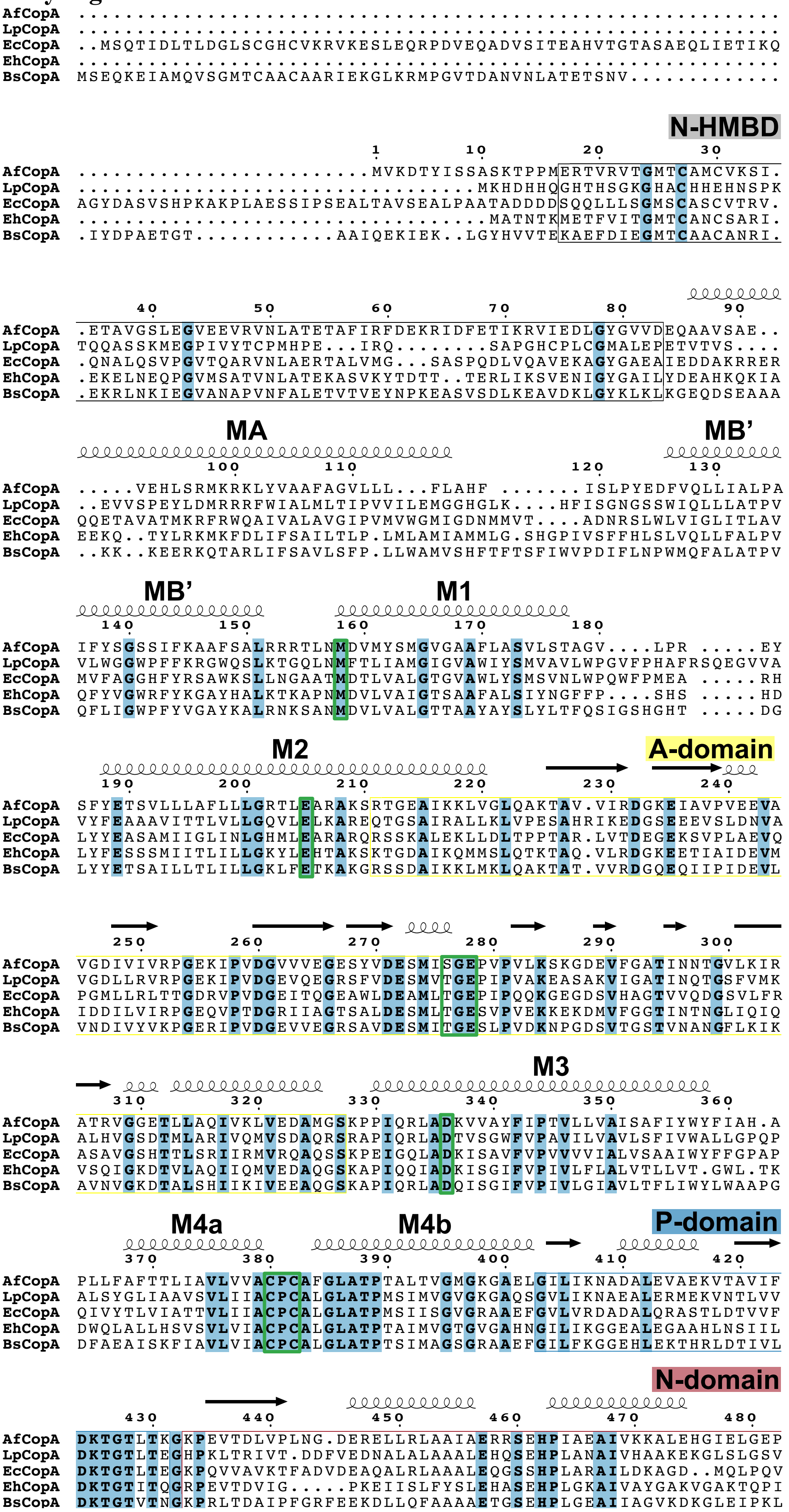

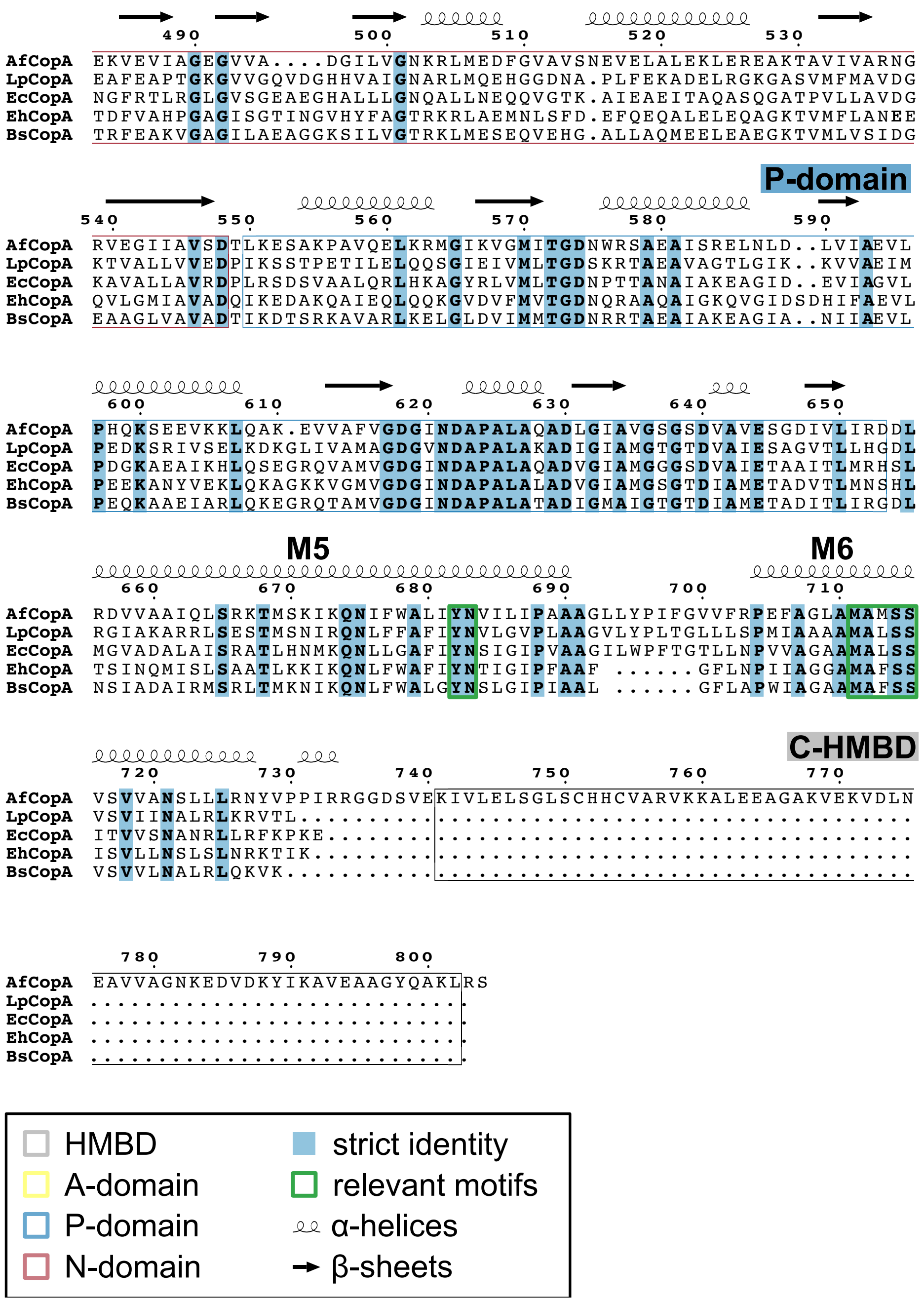

Supplementary Fig. 6. Multiple sequence alignment of bacterial CopA proteins. The multiple sequence alignment was performed using Clustal Omega [1] and visualized in ESPript [2]. Secondary structure features are shown based on the determined AfCopA structure. Transmembrane helices are labeled, and soluble domains are highlighted by colored boxes. Blue color indicates strict identity. Relevant motifs are stressed with green boxes.

Supplementary Fig. 7.

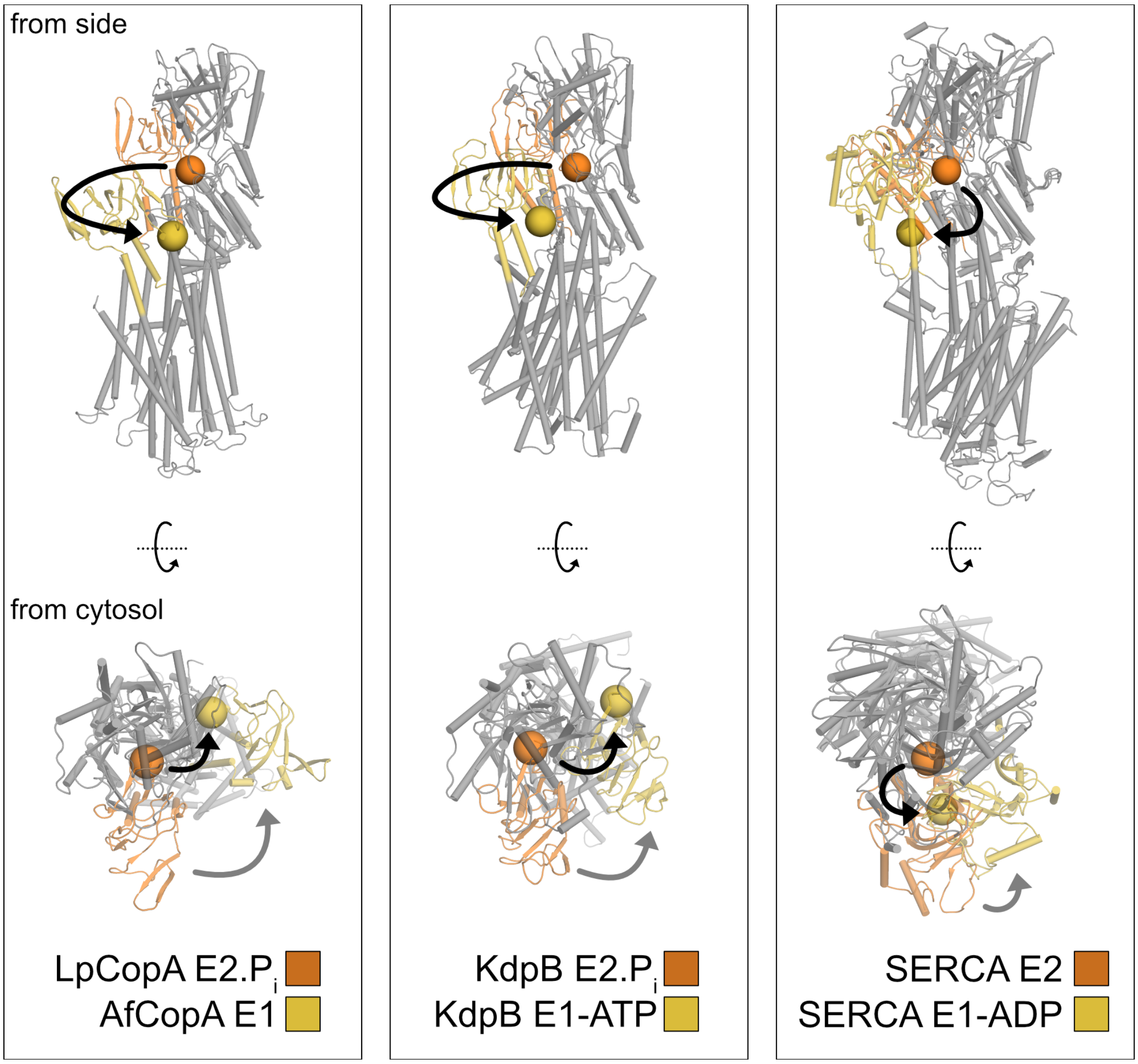

Supplementary Fig. 7. The A-domain rotation associated with the E2.P$_i$ → E1 transition. Structural alignment on the P-domain of the E1 and E2.P$_i$ states of P$_{1B}$- (CopA; AfCopA and LpCopA), P$_{1A}$- (KdpB) and P$_{2A}$-ATPases (SERCA). The A-domains are shown in orange and in yellow in the E2.P$_i$ and E1 states, respectively, with the (T/S)GE dephosphorylation loop highlighted as a sphere. The E1 state of both P$_{1A}$- and P$_{1B}$-ATPases is characterized by a large spatial distance between the A- and N-domain. During the E2.P$_i$ → E1 transition, the A-domain (grey arrow) and (T/S)GE motif (black arrow) rotate counter-clockwise as seen from the cytosol. In SERCA, the distal part (not facing the core of the protein) of the A-domain moves in a similar manner (grey arrow), whereas the TGE loop (black arrow) turns in the opposite direction. Highly homologous E1 structures of KdpB are available, determined in the absence or in the presence of nucleotide [3] The following PDB-IDs were employed: LpCopA E2.P$_i$ (3RFU), KdpB E2.P$_i$ (7BH2), KdpB E1-ATP (7LC3), SERCA E2 (3NAL), SERCA E1-ADP (1T5T).

Supplementary Fig. 8.

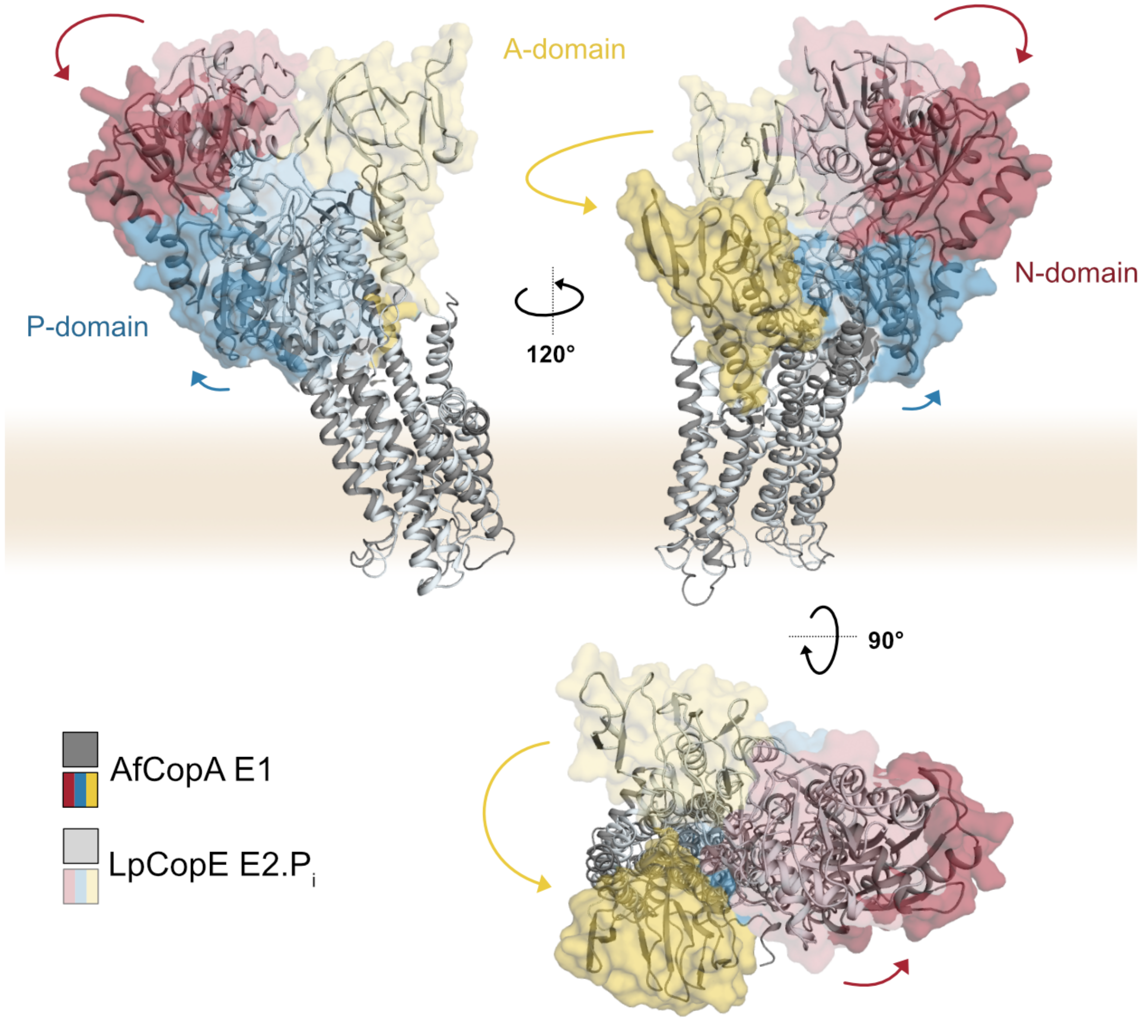

Supplementary Fig. 8. Soluble domain movements relative to the M-domain during the E2.Pᵢ → E1 transition. Alignment of the determined AfCopA E1 structure to transmembrane helices M3-6 of the LpCopA E2.Pᵢ state. Soluble domains are shown as surfaces.

Supplementary Fig. 9.

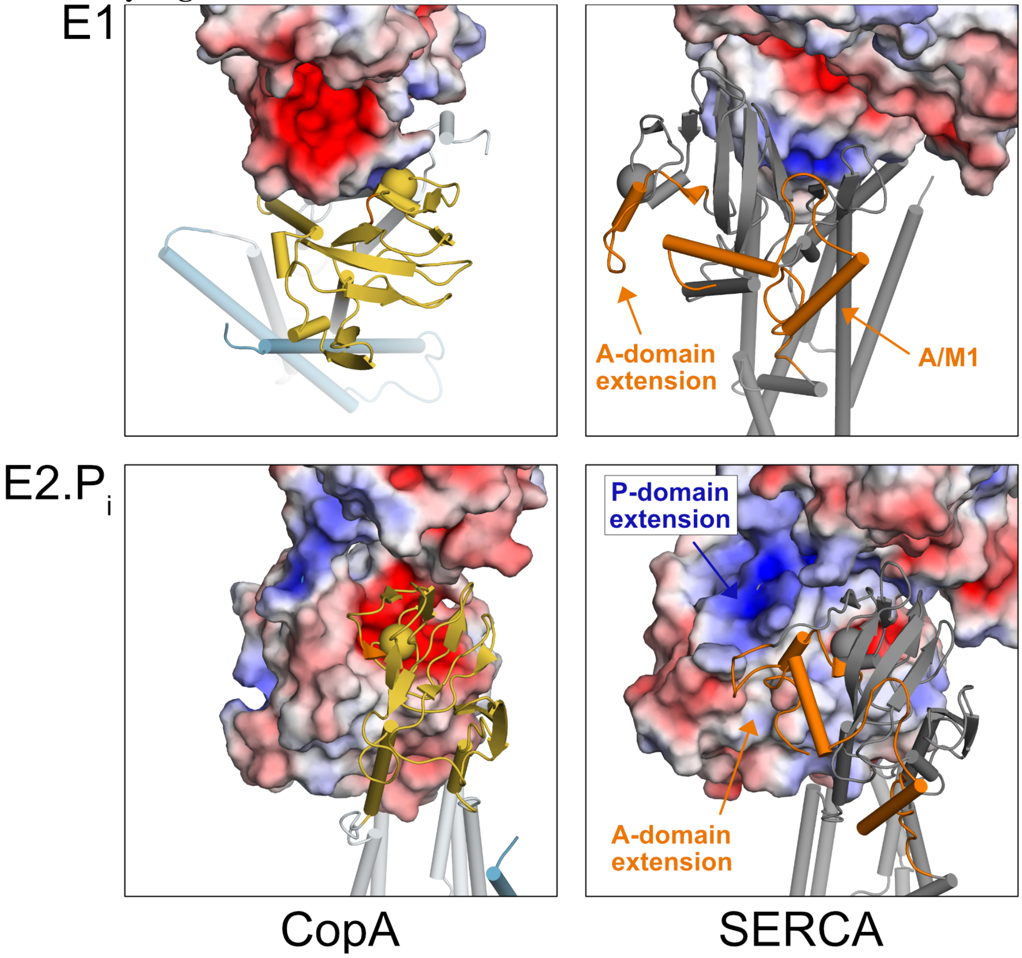

Supplementary Fig. 9. The interaction of the A-domain with the P-type ATPase core. The E1 and E2.P_i states of CopA proteins (AfCopA E1 determined in this work, and the E2.P_i state of LpCopA, PDB-ID 3RFU) are compared to the corresponding states of SERCA (PDB-IDs 4H1W and 3FGO). The surface charge of the P- and N-domains are shown, and the remaining parts of the proteins are displayed as cartoon (with CopA colored as in Fig. 1 and SERCA in grey). The A-domain extensions of SERCA are colored in orange. The TGE motifs are shown as spheres. In the E2.P_i state of SERCA, the A-domain extension is interacting with an electropositive extension of the P-domain, likely stabilizing this conformation. Both extensions are absent in CopA proteins.

Supplementary Fig. 10.

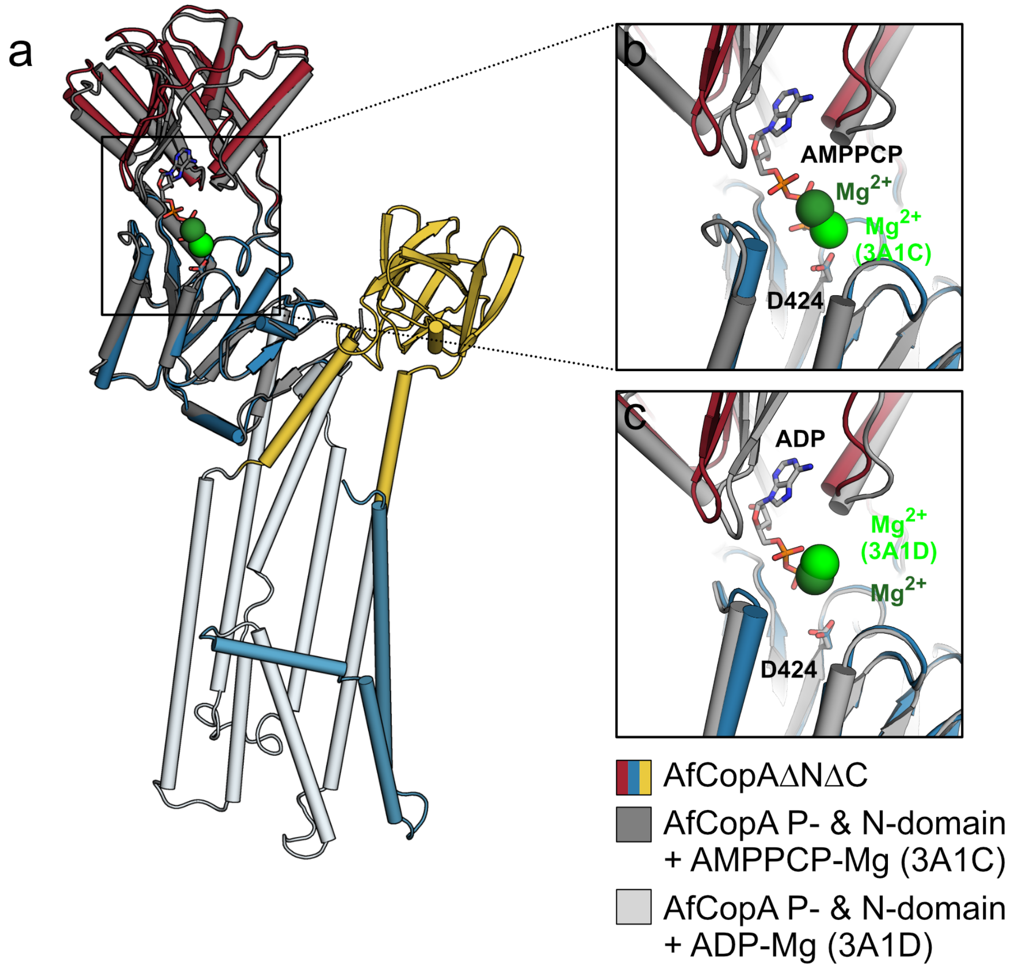

Supplementary Fig. 10. The nucleotide binding area. a Overview and structure-based alignment to the previously determined crystal structure of the P- and N-domains of AfCopA in complex with AMPPCP-Mg (PDB-ID 3A1C, dark grey) [4]. Structures are aligned on the P-domains. **b** Close-view showing the nucleotide binding area of the models shown in a. **c** Alignment to the crystal structure of the AfCopA P- and N-domains in complex with ADP-Mg (PDB-ID 3A1D, light grey) [4]. The N-domain of the determined AfCopA structure is slightly shifted compared to the nucleotide-bound structures of the isolated P- and N-domains, but the determined conformation is likely able to bind nucleotides.

Supplementary Fig. 11.

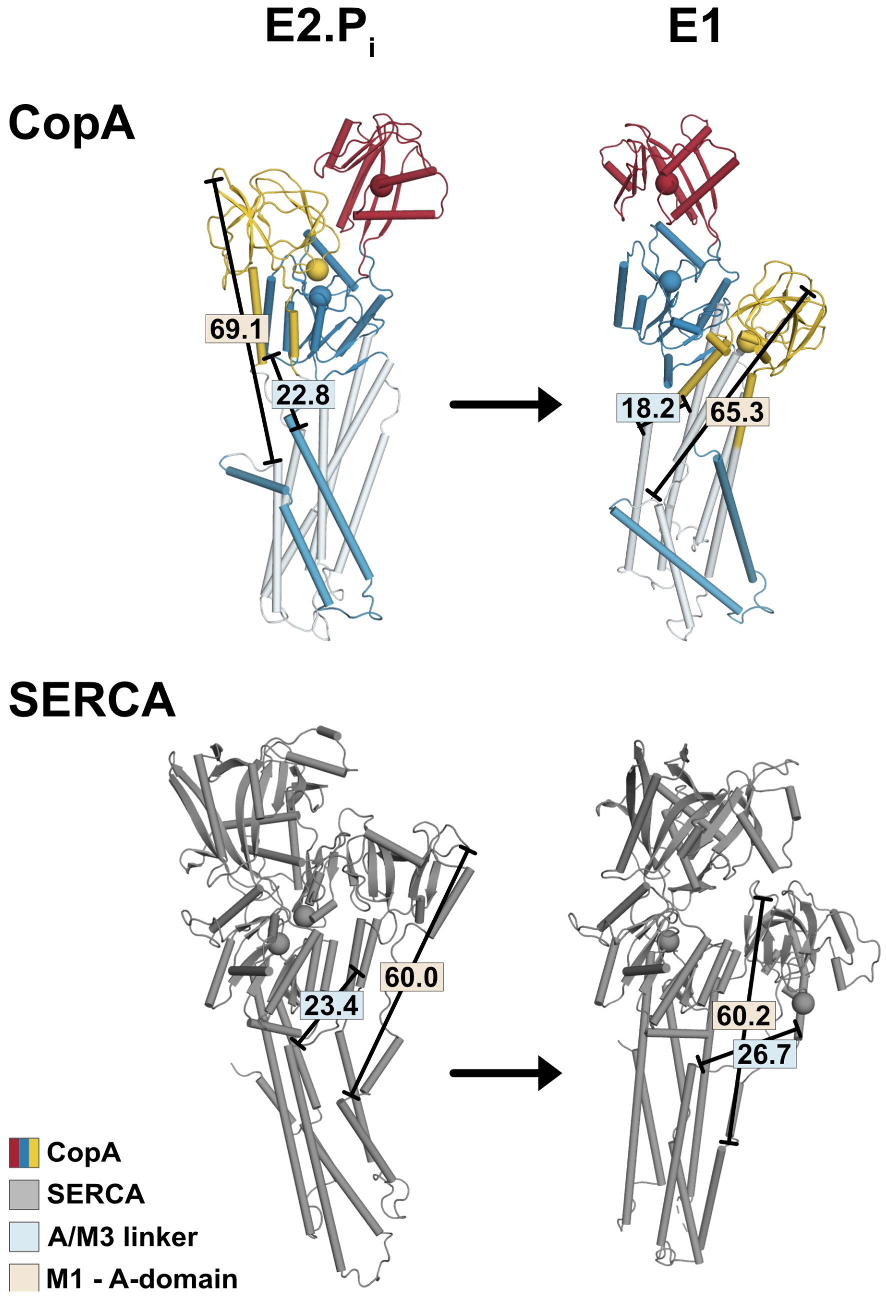

Supplementary Fig. 11. Change of domain distances during the E2.P$_i$ → E1 transition. The E1 and E2.P$_i$ states of CopA proteins are compared to the corresponding states of SERCA (AfCopA E1 as determined in this work, and PDB-IDs 3RFU, 4H1W, 3FGO). The length of the A/M3-linker is shown in rectangles with blue background, and the distance from the cytoplasmic end of M1 to the distal part of the A-domain in rectangles with orange background. During the E2.P$_i$ → E1 transition of SERCA, the distance from M1 to the distal part of the A-domain remains essentially unchanged, while it decreases in CopA proteins. Notably, the length of the A/M3-linker increases during the E2.P$_i$ → E1 transition in SERCA, but decreases in CopA proteins.

Supplementary Fig. 12.

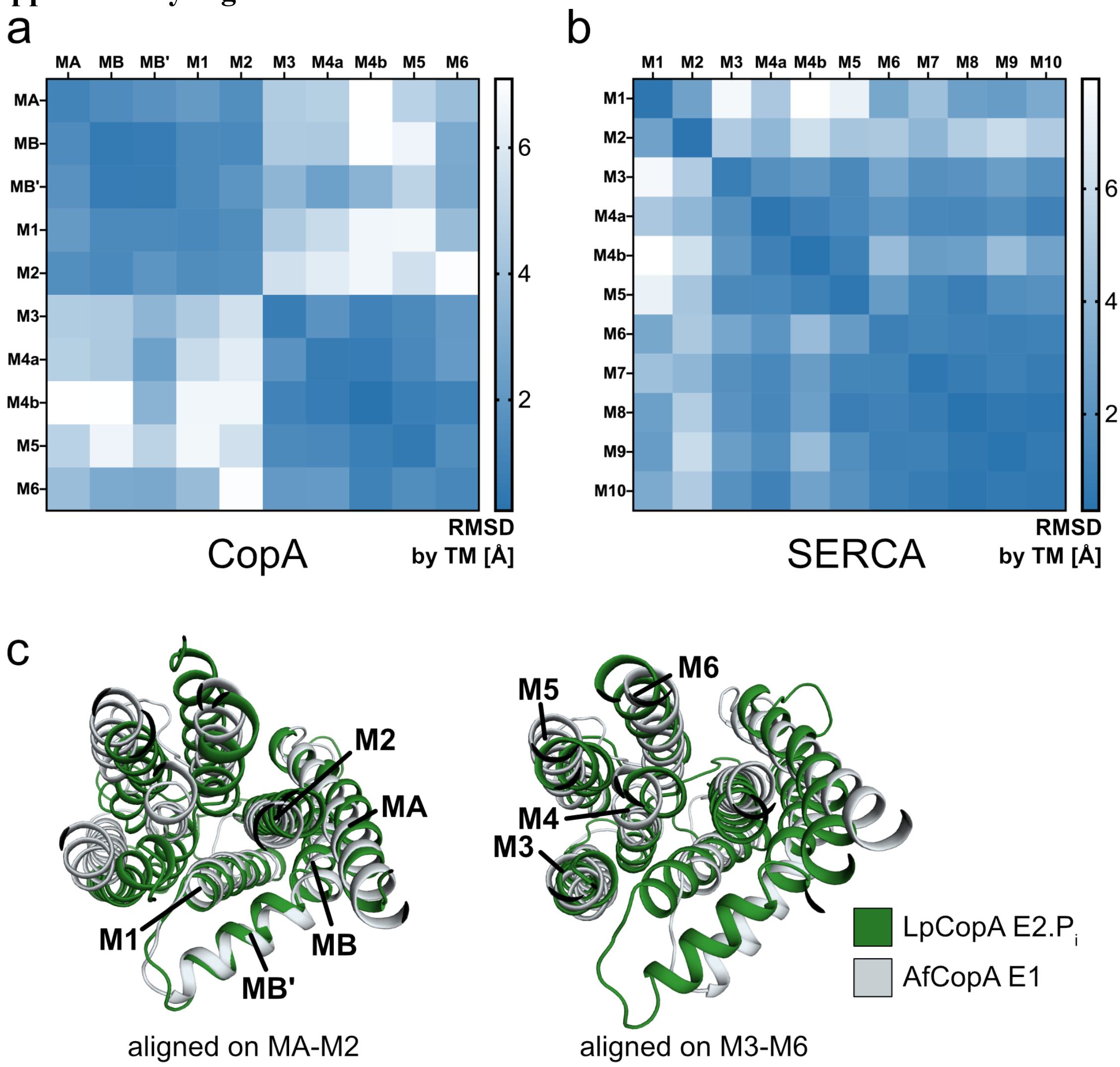

Supplementary Fig. 12. Rearrangements of the M-domain during the E2 → E1 transition. **a** Distance difference matrix (DDM) of the determined AfCopA structure as compared to the E2.P_i state of LpCopA (PDB-ID 3RFU). MA-M2 and M3-M6 form two helix bundles that move relative to each other in the E2.P_i → E1 transition. **b** Similar DDM for SERCA (PDB-ID 3FGO and 4H1W), where M7-10 appear rigid as previously proposed [5]. **c** Structural alignments of the determined AfCopA structure (green) to the E2.P_i structure of LpCopA (wheat), as viewed from the cytoplasm. Alignments based on MA-M2 or M3-M6 are shown.

Supplementary Fig. 13.

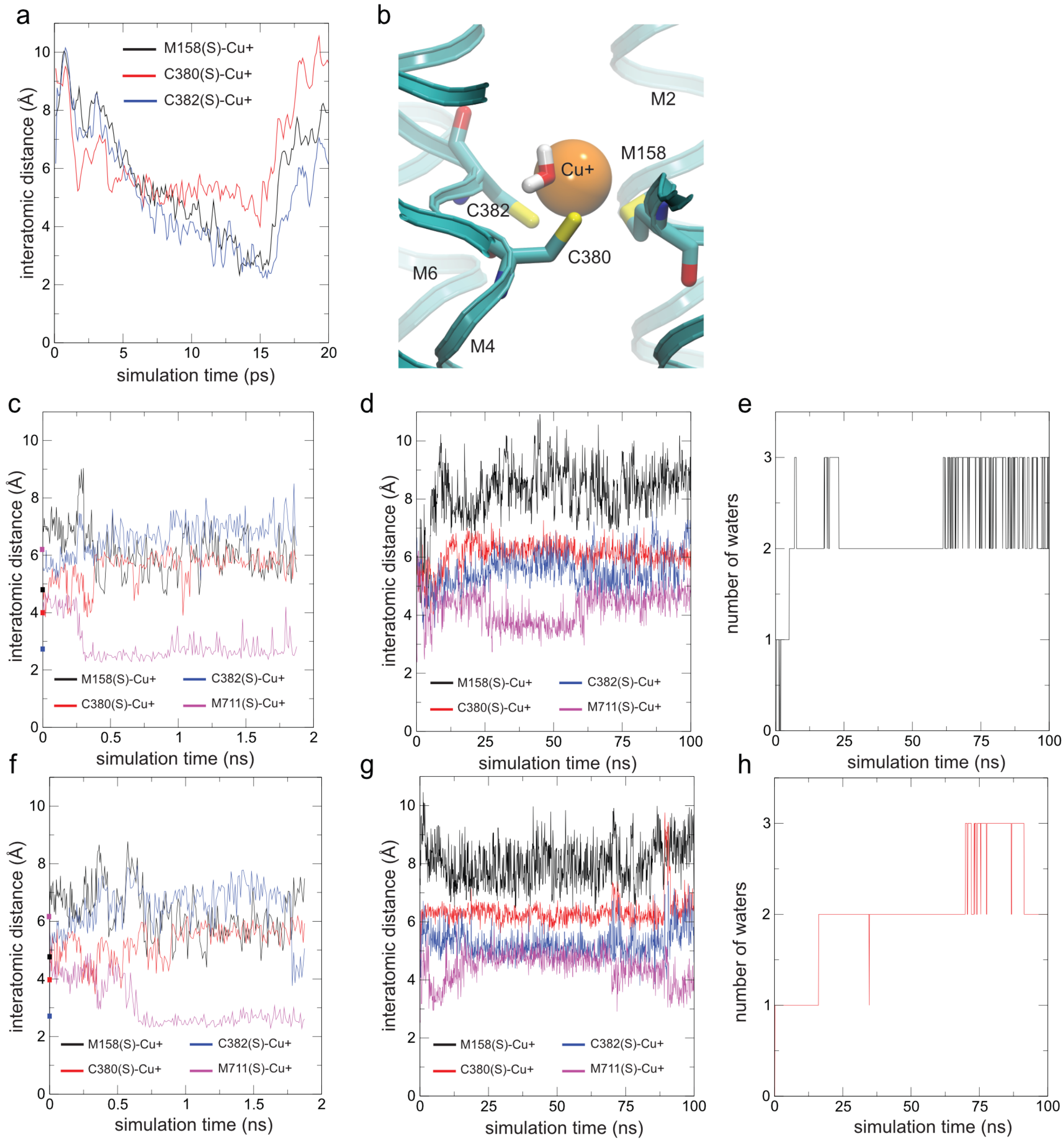

Supplementary Fig. 13. Simulated Cu^{+} coordination and entry pathway dynamics in AfCopA. Simulated interatomic distances between Cu^{+} and the sulfur atoms of M158 (black line), C380 (red line), C382 (blue line) or M711 (magenta line). **a** The simulated 20 ps trajectory, with Cu^{+} initially placed 5 Å away from the M158-C380-C382 entry site into the surrounding aqueous environment (as in Fig. 4b). **b** Simulation frame at 15 ps from the 20 ps trajectory depicting the trigonal coordination by residues M158, C380, and C382 to Cu^{+}, which also involves a water molecule in the C380 interaction. Following 15 ps the Cu^{+} continues further into the membrane, towards M711, see also panels c-h. **c,f** The initial 2 ns equilibration starting from the structure refined in the presence of Cu^{+} (as in Fig. 4d). **d,g** The corresponding subsequent 100 ns production runs based on panels c and f. **e,h** Number of water molecules within 3 Å from Cu^{+} in the simulation trajectories presented in panels d and g.

Supplementary Fig. 14.

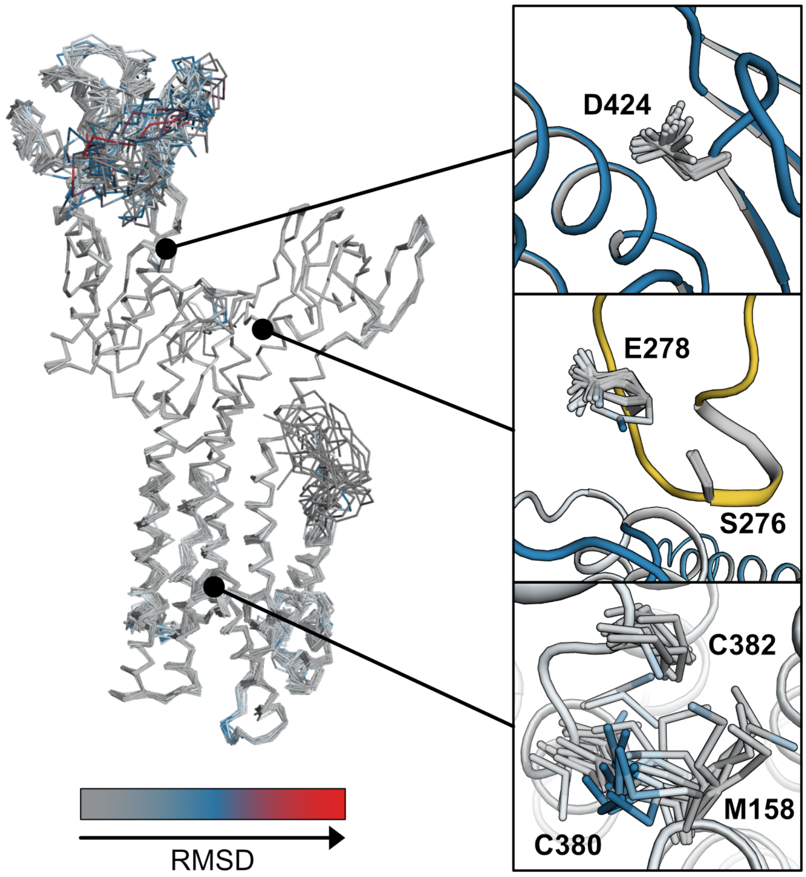

Supplementary Fig. 14. Ensemble Refinement. Ensemble refinement was performed as implemented in Phenix [6], using the refined high-resolution data set. Default settings were used. Ensemble Refinement yielded 18 ensembles, that were colored by Root Mean Square Deviation (RMSD) using colorbyrmsd.py. The A- and P-domains feature low RMSDs, while the N- and parts of the M-domain generated increased RMSD values. Insets show side chain conformations of all 18 ensembles, again colored by RMSD. Most side chains in the A- and P-domains show definite conformations (e.g. S276), while multiple side chain configurations were identified in the region around the copper entry site (e.g. C380 and M158).

Supplementary Fig. 15.

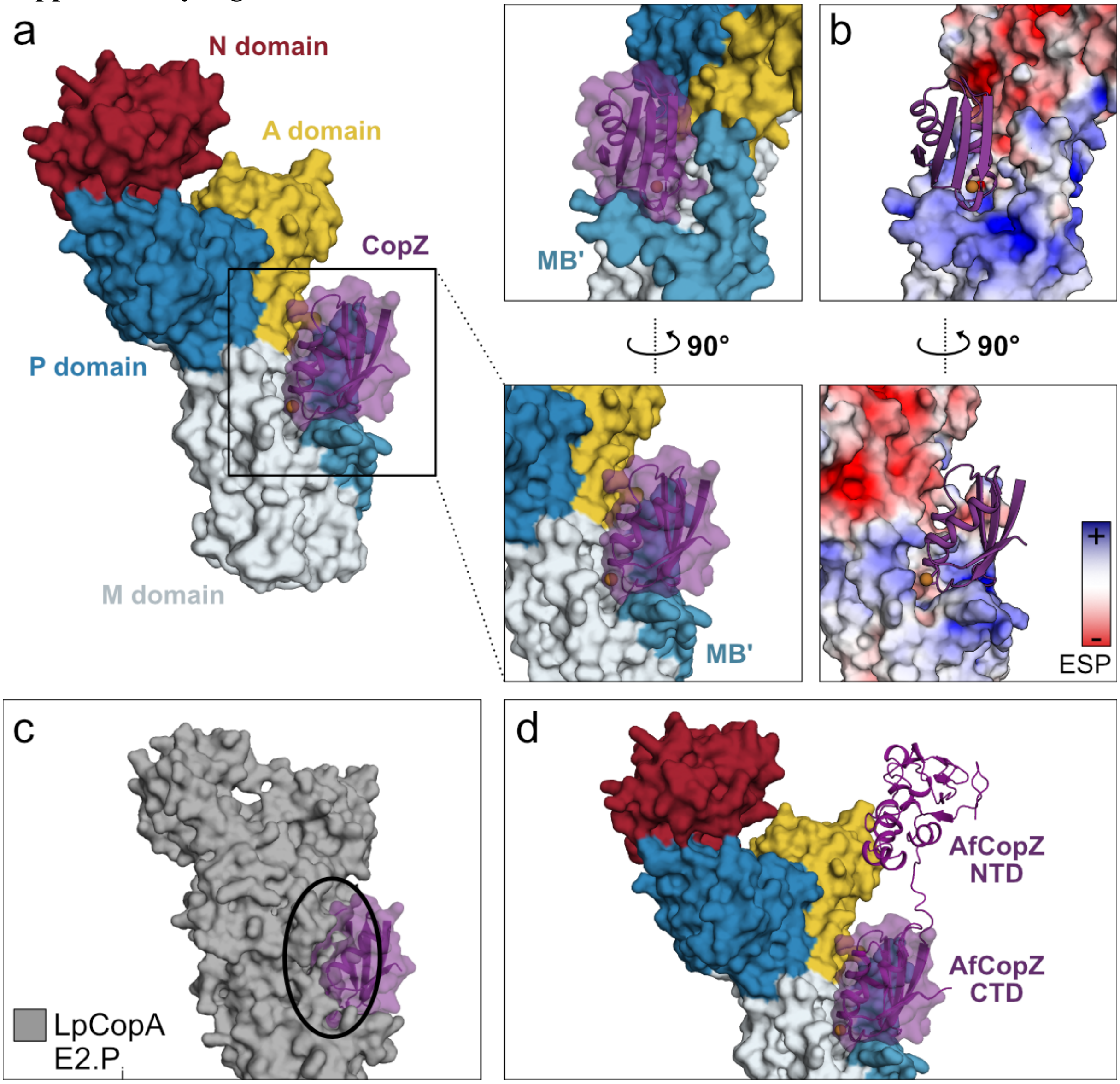

Supplementary Fig. 15. Docking of CopZ to the CopA E1 conformation. a A homology model of the CxxC-harboring C-terminal part of AfCopZ was generated in SwissModel [7] based on the *E. hirae* CopZ structure (PDB-ID 1CPZ) [8] as a template. Docking to the determined AfCopA E1 structure was performed using pyDockWEB [9]. CopA is colored as in Fig. 1 and CopZ in purple. **b** The surface charge of AfCopA is displayed using identical views as the insets of panel **a**, and CopZ is shown in purple. **c** CopZ docking site in the LpCopA E2.P$_i$ state. The LpCopA E2.P$_i$ structure was aligned to MA-MB' of AfCopA E1 state, illustrating that the E2.P$_i$ state does not provide sufficient space for CopZ binding to this site, as CopZ interferes with the E2.P$_i$ structure. **d** Docking model for full-length AfCopZ. AfCopZ contains an additional N-terminal domain (NTD), for which the above-mentioned docking model is compatible, as there is sufficient of space to permit the presence also of the NTD. The model for the full-length AfCopZ was downloaded from the AlphaFold Protein Structure Database (Uniprot ID 029901) [10].

Supplementary Fig. 16.

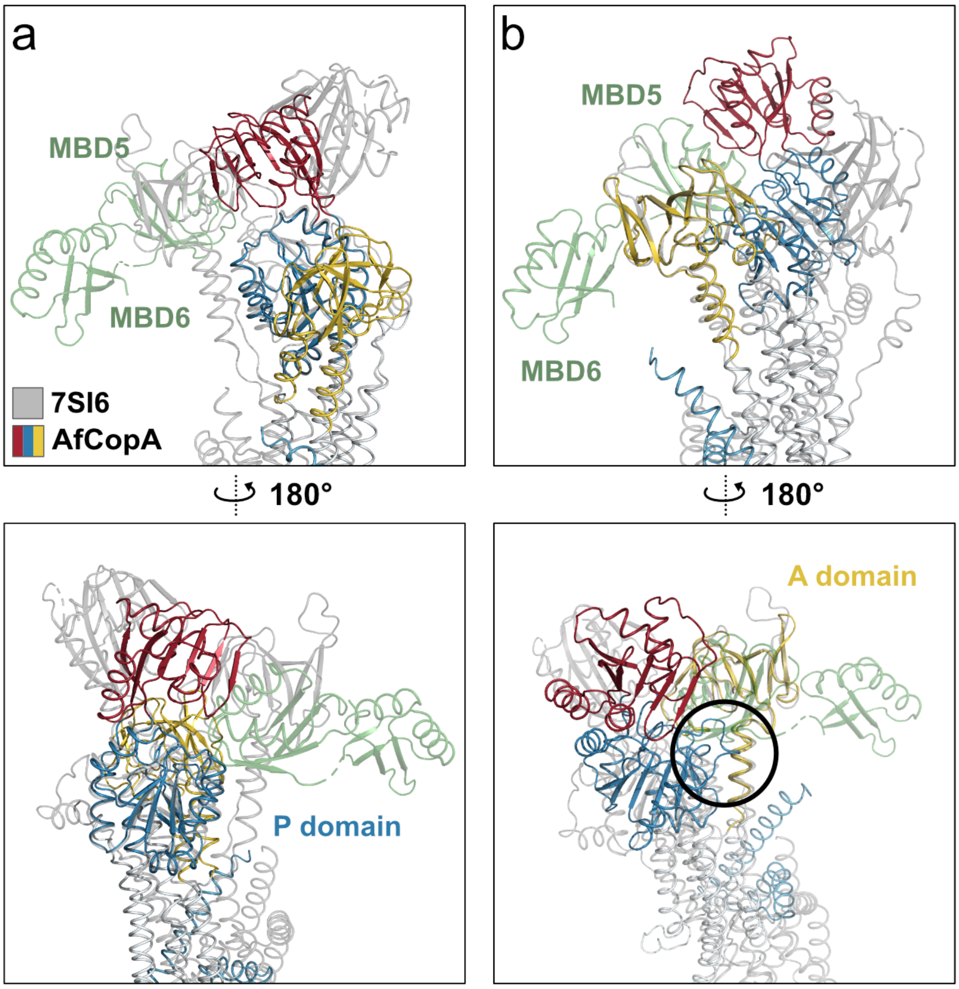

Supplementary Fig. 16. Alignment of the AfCopA E1 structure to the E2 state of frog ATP7B (PDB-ID 7SI6). The heavy metal binding domains (HMBDs) of frog ATP7B are shown in green. **a** If aligned on the P-domain, the AfCopA E1 state will provide sufficient space for HMBD binding in the same way as in the ATP7B E2 state. However, as the A-domain is significantly rotated in AfCopA E1 compared to ATP7B E2, no stabilizing interactions between the HMBD and the A-domain are possible in the E1 conformation, thereby precluding inhibition. **b** If aligned on the A-domain, there will not be sufficient space for HMBD binding to AfCopA, as the P-domain of AfCopA E1 will overlap with the HMBD of frog ATP7B, thereby precluding inhibition (highlighted with circle).

Supplementary Fig. 17.

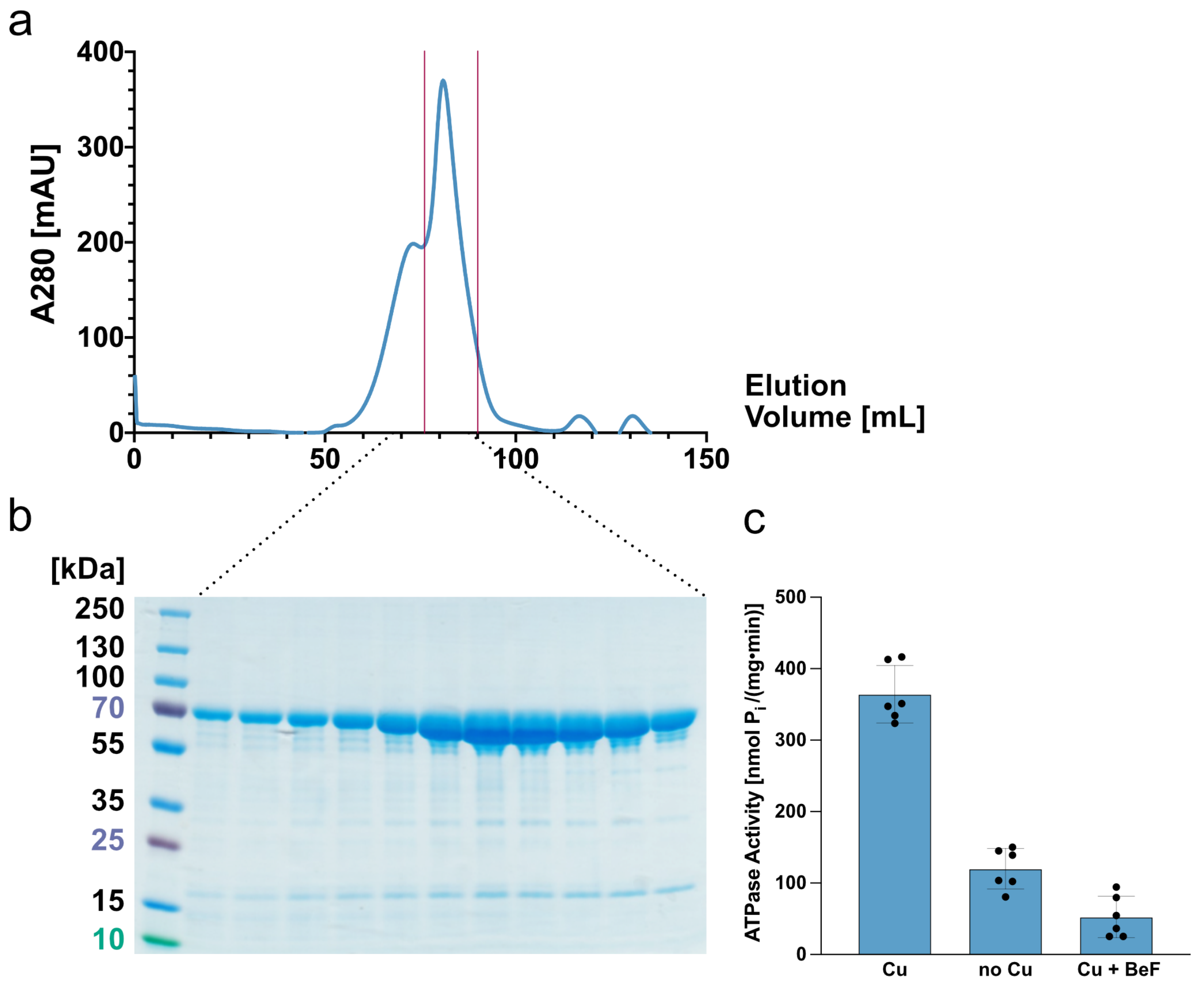

Supplementary Fig. 17. Purification and ATPase activity assay. a Size-exclusion chromatography of AfCopAΔNΔC using a Superose 6 resin column (see Methods for details). The red highlighted fractions were pooled, concentrated and used for crystallization. **b** Coomassie-stained SDS-PAGE of the sample shown in panel **a**. The molecular weight of AfCopAΔNΔC is 72.5 kDa. **c** ATPase activity assay. 30 % background activity was detected in the absence of Cu^+. The addition of the P-type ATPase inhibitor BeF abolished Cu^+-stimulated ATPase activity. Data from two biologically independent samples and n=6 independent experiments are presented as mean ± SD. Source data are provided as a Source Data file.

SUPPLEMENTARY REFERENCES

1. Madeira, F. *et al.* The EMBL-EBI search and sequence analysis tools APIs in 2019. *Nucleic Acids Res.* **47**, W636–W641 (2019).
2. Robert, X. & Gouet, P. Deciphering key features in protein structures with the new ENDscript server. *Nucleic Acids Res.* **42**, W320–W324 (2014).
3. Sweet, M. E. *et al.* Structural basis for potassium transport in prokaryotes by KdpFABC. *Proc. Natl. Acad. Sci. U.S.A.* **118**, e2105195118 (2021).
4. Tsuda, T. & Toyoshima, C. Nucleotide recognition by CopA, a Cu+-transporting P-type ATPase. *EMBO J* **28**, 1782–1791 (2009).
5. Møller, J. V., Olesen, C., Winther, A.-M. L. & Nissen, P. The sarcoplasmic Ca $^{2+}$ -ATPase: design of a perfect chemi-osmotic pump. *Quart. Rev. Biophys.* **43**, 501–566 (2010).
6. Burnley, B. T., Afonine, P. V., Adams, P. D. & Gros, P. Modelling dynamics in protein crystal structures by ensemble refinement. *Elife* **1**, e00311 (2012).
7. Waterhouse, A. *et al.* SWISS-MODEL: homology modelling of protein structures and complexes. *Nucleic Acids Research* **46**, W296–W303 (2018).
8. Wimmer, R., Herrmann, T., Solioz, M. & Wüthrich, K. NMR structure and metal interactions of the CopZ copper chaperone. *J Biol Chem* **274**, 22597–22603 (1999).
9. Jiménez-García, B., Pons, C. & Fernández-Recio, J. pyDockWEB: a web server for rigid-body protein–protein docking using electrostatics and desolvation scoring. *Bioinformatics* **29**, 1698–1699 (2013).
10. Jumper, J. *et al.* Highly accurate protein structure prediction with AlphaFold. *Nature* **596**, 583–589 (2021).

Uncropped version of the SDS-PAGE gel in Supplementary Fig. 17b.

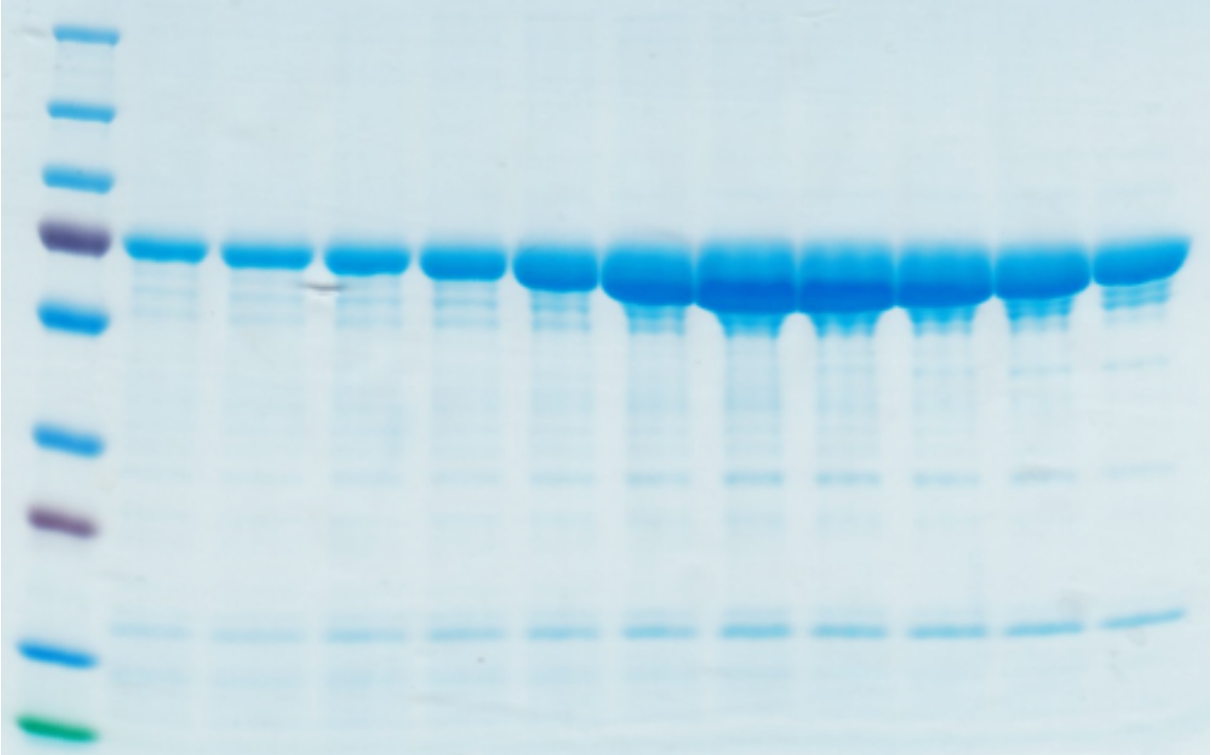

Supplement: Supplementary file 1 — Supplementary Information [file 41467_2022_32751_MOESM1_ESM.pdf]
