# Supplementary material for: Structural basis of ion uptake in copper-transporting P1B-type ATPases
Source: Nat Commun. 2022 Aug 31;13:5121. doi: 10.1038/s41467-022-32751-w (PMC9433437; doi:10.1038/s41467-022-32751-w)
Supplement: Supplementary file 3 — Reporting Summary [file 41467_2022_32751_MOESM3_ESM.pdf]

nature research

Corresponding author(s): Pontus Gourdon

Last updated by author(s): Aug 01, 2022

Reporting Summary

Nature Research wishes to improve the reproducibility of the work that we publish. This form provides structure for consistency and transparency in reporting. For further information on Nature Research policies, see our Editorial Policies and the Editorial Policy Checklist.

Statistics

For all statistical analyses, confirm that the following items are present in the figure legend, table legend, main text, or Methods section.

n/a	Confirmed	
☐	☒	The exact sample size (*n*) for each experimental group/condition, given as a discrete number and unit of measurement
☐	☒	A statement on whether measurements were taken from distinct samples or whether the same sample was measured repeatedly
☒	☐	The statistical test(s) used AND whether they are one- or two-sided<br>*Only common tests should be described solely by name; describe more complex techniques in the Methods section.*
☒	☐	A description of all covariates tested
☒	☐	A description of any assumptions or corrections, such as tests of normality and adjustment for multiple comparisons
☐	☒	A full description of the statistical parameters including central tendency (e.g. means) or other basic estimates (e.g. regression coefficient) AND variation (e.g. standard deviation) or associated estimates of uncertainty (e.g. confidence intervals)
☒	☐	For null hypothesis testing, the test statistic (e.g. *F*, *t*, *r*) with confidence intervals, effect sizes, degrees of freedom and *P* value noted<br>*Give P values as exact values whenever suitable.*
☒	☐	For Bayesian analysis, information on the choice of priors and Markov chain Monte Carlo settings
☒	☐	For hierarchical and complex designs, identification of the appropriate level for tests and full reporting of outcomes
☒	☐	Estimates of effect sizes (e.g. Cohen's *d*, Pearson's *r*), indicating how they were calculated

Our web collection on statistics for biologists contains articles on many of the points above.

Software and code

Policy information about availability of computer code

Data collection	X-ray crystallography data sets were collected at Swiss Light Source, the Paul Scherrer Institute, Villigen, Switzerland, beam line X06SA
Data analysis	X-ray crystallographic data were processed and scaled using XDS, build 20200131. Downstream structure determination and refinement was accomplished using Phenix 1.19 and coot 1. Functional data were analyzed and visualized using Graph Pad Prism 9. All structural figures were generated using PyMOL 2.5.1. Computational docking was performed using pyDockWEB (online). Alignments were performed using Clustal Omega (online), and visualized using ESPript 3.0.

For manuscripts utilizing custom algorithms or software that are central to the research but not yet described in published literature, software must be made available to editors and reviewers. We strongly encourage code deposition in a community repository (e.g. GitHub). See the Nature Research guidelines for submitting code & software for further information.

Data

Policy information about availability of data

All manuscripts must include a data availability statement. This statement should provide the following information, where applicable:

- Accession codes, unique identifiers, or web links for publicly available datasets
- A list of figures that have associated raw data
- A description of any restrictions on data availability

The structural coordinates generated in this study have been deposited in the Protein Data Bank under accession codes: 7R0I [http://doi.org/10.2210/pdb7R0I/pdb] (E1 in the presence of copper), 7R0H [http://doi.org/10.2210/pdb7R0H/pdb] (E1 in the presence of copper with data collected at the copper edge), 7R0G [http://doi.org/10.2210/pdb7R0G/pdb] (E1 in the absence of copper). Source data are provided with this paper. All data and materials supporting the findings in the manuscript are available from the corresponding author upon reasonable request.

Field-specific reporting

Please select the one below that is the best fit for your research. If you are not sure, read the appropriate sections before making your selection.

☒ Life sciences ☐ Behavioural & social sciences ☐ Ecological, evolutionary & environmental sciences

For a reference copy of the document with all sections, see nature.com/documents/nr-reporting-summary-flat.pdf

Life sciences study design

All studies must disclose on these points even when the disclosure is negative.

Sample size	Complete X-ray crystallography statistics are provided in the Methods section and Supplementary Table 1. Sample size determination was not used for the ATPase activity and ICP-MS measurements. The sample size was selected based on the standard in the literature (three independent experiments)
Data exclusions	Complete X-ray crystallography statistics are provided in the Methods section and Supplementary Table 1.
Replication	AfCopAdNdC was produced and purified multiple times. All productions and purifications were successful. Multiple crystals were obtained from different purifications and screened. Data was collected for several crystals, and the crystals diffracting to highest resolution were used for further analysis. AfCopA mutants were produced and purified twice (independently on different days). For the ATPase activity and ICP-MS measurements, two or three independent measurements were performed on two biological replicates each, and all data are included in the analysis. For AfCopAdNdC, ATPase activity measurements were successfully repeated multiple times. No further attempts were performed for ATPase activity and ICP-MS experiments of AfCopA mutants.
Randomization	Samples for the biochemical assays were not randomized, as all samples were treated in an identical manner.
Blinding	No blinding was applied as no group allocation was used.

Reporting for specific materials, systems and methods

We require information from authors about some types of materials, experimental systems and methods used in many studies. Here, indicate whether each material, system or method listed is relevant to your study. If you are not sure if a list item applies to your research, read the appropriate section before selecting a response.

Materials & experimental systems

n/a	Involved in the study	
☒	☐	Antibodies
☒	☐	Eukaryotic cell lines
☒	☐	Palaeontology and archaeology
☒	☐	Animals and other organisms
☒	☐	Human research participants
☒	☐	Clinical data
☒	☐	Dual use research of concern

Methods

n/a	Involved in the study	
☒	☐	ChIP-seq
☒	☐	Flow cytometry
☒	☐	MRI-based neuroimaging
